# What size and shape am I?

**DOI:** 10.3389/fpsyg.2026.1946881

**Published:** 2026-09-28

**Authors:** Lise Gulli Brokjøb, Katri K. Cornelissen, Kjersti Lillevoll, Michael C. Grugan, Kristofor McCarty, Martin J. Tovée, Piers L. Cornelissen

**Affiliations:** 1 Department of Psychology, UiT - The Arctic University of Norway, Tromsø, Norway; 2 Department of Psychology, Northumbria University, Newcastle upon Tyne, United Kingdom; 3 Centre for Population Health, Haukeland University Hospital, Bergen, Norway

**Keywords:** attitudinal body image, body size, interoception, network analysis, perceptual body image, contraction bias, body size estimation

## Abstract

**Background:**

Body image disturbance is conceptualised as arising from the interaction between perceptual and attitudinal components, yet the psychological complexity embedded within the attitudinal domain remains underexplored. This study tested an integrated model of perceptual, attitudinal, and interoceptive influences on women’s judgments of their own body size.

**Methods:**

In a sample of 269 adult women, behavioural measures of body size estimation were combined with network analysis, exploratory factor analysis, and regression modelling to distinguish psychological processes exerting direct versus indirect effects on self-estimated body size.

**Results:**

Three central findings emerged. First, contraction bias in perceptual body size estimation was robustly replicated, with lower-BMI individuals overestimating and higher-BMI individuals underestimating their body size. Second, attitudinal body image concerns independently shifted self-estimated body size upward across the BMI range, acting additively rather than interactively with contraction bias. Third, the network and factor analyses demonstrated that attitudinal body image comprised four interconnected domains: Body Image, Mood, Interoceptive Sensibility, and Sociocultural Pressure. We found important connections between Body Image and Interoceptive Sensibility facilitated by Mood acting as a bridging intermediary. However, the regression analysis showed that only Body Image, not Interoceptive Sensibility, Sociocultural Pressure, or Mood, exerted direct effects on self-estimates of body size.

**Conclusion:**

Attitudinal body image is multidimensional in structure, but only the Body Image domain directly influences behavioural judgments of body size; interoceptive sensibility and other attitudinal factors operate indirectly within the broader body image system. The two-component perceptual–attitudinal model remains a powerful framework for explaining body size judgments, while network methods clarify how the contributing psychological processes are organised.

## 1 Introduction

### 1.1 Body image and body image disturbances

Body image, the way individuals perceive, evaluate, and relate to their own bodies, is a fundamental determinant of mental health and wellbeing (Gillen, 2015; Modica and Markley, 2024). Dissatisfaction with one’s body image leads to over-estimation of one’s body size, a phenomenon that is so common amongst otherwise healthy individuals in Western societies, that it has been dubbed normative discontent (Lacroix et al., 2023). Moreover, across both clinical and population-based studies, higher body image dissatisfaction is consistently linked to lower life satisfaction, anxiety, and depression (Cash and Smolak, 2011; Neumark-Sztainer et al., 2006), as well as increased risk for the onset, severity, and relapse of eating disorders such as anorexia nervosa (AN, Keel et al., 2005). While there is evidence to suggest that women with AN under-estimate their body size (Meermann, 1983), or even show performance in size estimation tasks equivalent to controls without eating disorders (Fernández et al., 1994; Meermann, 1983), most studies have found that patients with AN overestimate their body size (Gardner and Bokenkamp, 1996; Probst et al., 1998; Tovée et al., 2003). This fluidity of body size estimation in healthy and eating-disordered individuals raises a key question. In the absence of access to bathroom scales and objective measurement, how do we assess our body size?

### 1.2 Additive effects of attitudinal and perceptual body image

The conceptualization of body image by Cash and Deagle (1997) proposes separate and independent attitudinal and perceptual dimensions. This framework is useful for current purposes and has direct empirical support (Cornelissen et al., 2019). It leads to the assertion that a complete account of the question “what size do I think my body is?” necessarily requires summing information from both the perceptual and attitudinal domains.

To investigate the additive effects of attitudinal and perceptual body image in previous studies, healthy adult women carried out visual tasks in which they were presented CGI images of a female avatar ranging in BMI from 12.5 to 44.5 units (Brokjøb et al., 2024; Cornelissen et al., 2015, 2017). Participants identified the image closest to their perceived body size, from which self-estimated BMI was calculated. They also completed psychometric measures of body attitude, including assessments of eating disorder symptoms, body shape concerns, depression, self-esteem, and sociocultural pressures towards thinness. Moderate to large correlations between the psychometric measures were found across all three studies, so they were reduced via principal component analysis to a single component, PSYCH (expressed as *z*-scores). High positive PSYCH scores represent extensive psychological concerns with body shape, weight, and eating, low mood, and high internalisation of sociocultural pressures to be thin; low negative scores represent the opposite to this.

Multiple regression analyses revealed significant and independent influences of both actual BMI and PSYCH on self-estimated BMI as illustrated in Figure 1. No interactions were found between actual BMI and PSYCH in any study.

**Figure 1:**
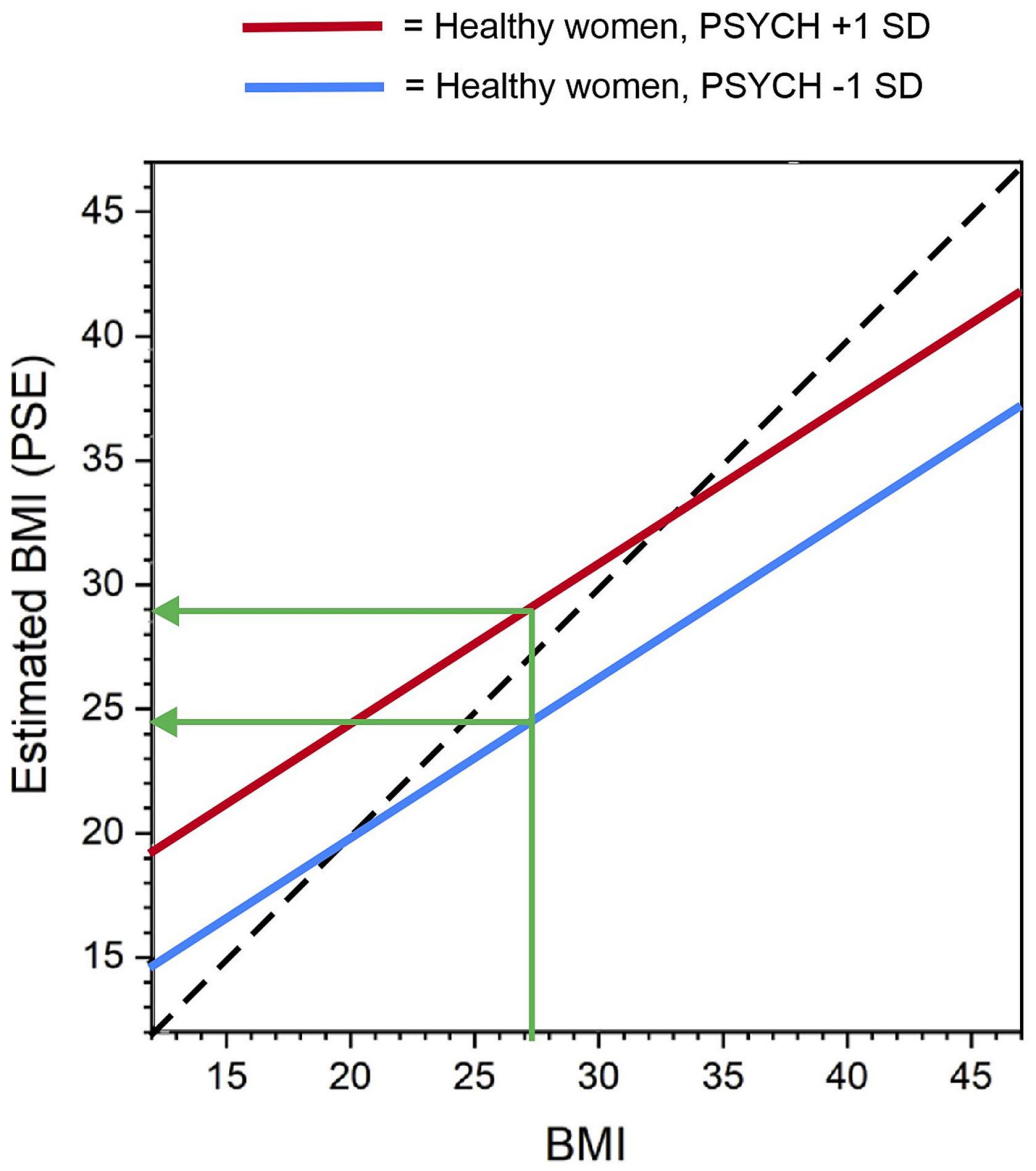
Sketch plot to illustrate findings by Cornelissen et al. (2015). Self-estimated body size is plotted as a function of actual body size (BMI). Red and blue lines represent PSYCH values at +1 SD and −1 SD, respectively (see text for details).

Figure 1 plots self-estimated body size against actual body size, with a black short-dashed line of equivalence (slope = 1) for reference. Healthy controls show regression slopes less than 1: those with BMIs near the population average estimated most accurately, while lower BMIs were over-estimated and higher BMIs under-estimated. This reflects contraction bias, a well-established, adaptive pattern in magnitude estimation found across multiple sensory domains and species (cf. Jannasch et al., 2025; Martynov et al., 2020; Turnbull et al., 2022; Wignall et al., 2017). Recent computational evidence supports a Bayesian framework in which contraction bias, alongside scalar and sequential biases, represents a near-optimal strategy for accurate magnitude estimation under sensory uncertainty, enhancing survival and reproductive success (Jannasch et al., 2025; Petzschner et al., 2015). The independent influence of PSYCH is shown by parallel regression lines at +1 and −1 PSYCH. For any given actual BMI, higher PSYCH scores produced larger self-estimates of body size.

### 1.3 The central problem

The essential point about these results is that information about both attitudinal body image (indexed by PSYCH, reflected in regression line intercepts in the above figure) and perceptual body image (reflected in regression line slopes) is required for a full account of participants’ self-estimates of body size, as observed empirically. This is emphasized in Figure 1 by the vertical green line from a single value for actual BMI. This line’s upward projection to intersect with the 2 regression lines produces 2 separate self-estimates of BMI. Thus, 2 individuals who have the same actual BMI each make different self-estimates of their body size (expressed in BMI units).

However, while this two-component model of body size estimation is well validated, replicable in women and men, and intuitively visualizable (see, e.g., Cornelissen et al., 2022; Groves et al., 2023; Gumančík et al., 2024), it may nevertheless be incomplete. Specifically, it collapses a wide range of interrelated psychological processes (mood, self-esteem, sociocultural pressures, eating pathology, body image concern) into a single index (represented by PSYCH in Figure 1). This raises an important conceptual question: what is being lost when attitudinal body image is treated as such a unitary construct? In short, would an expanded set of attitudinal body image metrics improve our ability to explain self-estimates of body size and shape. A more nuanced analytic approach is therefore needed to examine the adequacy of this simplistic two-component model. We need to explore the complexity of attitudinal factors and their potential influence on self-estimates of body size in a more fine-grained way. One candidate domain that may be particularly relevant, and that has not been included in previous models, is interoceptive sensibility.

### 1.4 A role for interoceptive sensibility in body size judgement?

Interoception refers to sensing internal bodily signals. Current theory distinguishes several different features; interoceptive accuracy (objective performance, such as heart-beat counting), interoceptive sensibility (self-reported tendencies to notice and value bodily sensations), and metacognitive insight about accuracy (Khalsa and Lapidus, 2016; Khalsa et al., 2018). Interoceptive sensibility is typically assessed with questionnaires such as the Multidimensional Assessment of Interoceptive Awareness (MAIA), which separates adaptive facets (i.e., the flexible, context-sensitive ways internal body signals are interpreted to regulate behaviour) from hypervigilant or dysregulated noticing (i.e., where bodily sensations are excessively monitored and amplified) (Mehling et al., 2018; Garfinkel et al., 2015).

Across clinical and nonclinical samples, atypical interoceptive processing is consistently linked to body image disturbance. Individuals with eating disorders (EDs) show reduced interoceptive accuracy on heartbeat-detection tasks and endorse more interoceptive difficulties on self-report measures derived from the Eating Disorder Inventory (Pollatos et al., 2008; Jenkinson et al., 2018). It is suggested that interoception is a significant contributor to disrupted embodiment across EDs, body dysmorphic disorder, and related conditions (Badoud and Tsakiris, 2017; Khalsa et al., 2018; Quadt et al., 2018).

Functionally, interoceptive signals appear to stabilize body representations by providing internally generated constraints that are integrated with exteroceptive cues (vision, touch, proprioception). For example, people with higher interoceptive accuracy are less susceptible to the rubber hand illusion, suggesting reduced malleability of body ownership under visuo-tactile conflict (Tsakiris et al., 2011). Eating disorder traits are associated with greater illusion susceptibility, consistent with weaker interoceptive constraints (Eshkevari et al., 2012). Interoceptive-exteroceptive integration can also be manipulated directly: synchronizing visual feedback with the heartbeat alters self-identification with a seen body, showing that cardiac interoceptive cues causally shape embodiment (Suzuki et al., 2013).

How does this affect perceived body size? Experimental body-ownership and body-size illusions demonstrate that multisensory conflicts can rapidly shift the perceived size of the body or body parts. For example, embodying scaled bodies (smaller or larger than one’s own) reliably rescales perceived bodily metrics and even the perceived size of objects in the environment (van der Hoort et al., 2011). Other paradigms show that illusory changes in body-part size modulate body satisfaction and related judgments (e.g., “feeling thinner” or “larger”) (Preston and Ehrsson, 2014). Because individual differences in interoceptive ability predict the malleability of body representations, the best-supported inference is that interoceptive processes influence how easily perceived body size can be biased by conflicting sensory evidence. In other words, stronger interoceptive signals (and/or more adaptive interoceptive sensibility) are thought to minimise visually driven size distortions, whereas weaker or dysregulated interoception allows visual priors and contextual cues to exert a larger effect.

Direct, correlational links between interoceptive sensibility specifically (self-reported) and the absolute body size people believe they have are limited and somewhat mixed and somewhat mixed. Several cross-sectional studies report that more adaptive interoceptive sensibility is associated with more positive body image, less body checking, and lower drive for thinness, whereas hypervigilant noticing without regulatory capacity tracks greater body dissatisfaction (Herbert et al., 2013; Badoud and Tsakiris, 2017). In clinical contexts, measures of interoceptive difficulties co-vary with overestimation of body size and with disturbance of body-scaled actions (e.g., overly cautious judgments about fitting through apertures) in anorexia nervosa, although these studies more often index interoceptive accuracy than sensibility (Pollatos et al., 2008; Keizer et al., 2013). Overall, when associations with perceived size are reported, effect sizes tend to be small-to-moderate and can depend on which facet of sensibility is probed (e.g., trusting vs. hypervigilant noticing), the task used to estimate body size (e.g., digital morphing, affordance judgments, VR self-avatars), and whether objective interoceptive accuracy is controlled.

In summary, interoceptive processing is robustly implicated in body image. However, the strongest evidence that it influences perceived body size is only indirect: better interoceptive processing reduces the malleability of body representations under multisensory conflicts that commonly shift perceived size, whereas weaker/dysregulated interoception is associated with greater susceptibility to such distortions. Therefore, in the current study we measured women’s estimated body size directly, using a figure rating scale configured as a sensitive method of adjustment task (MoA). We also included interoceptive sensibility as part of an extended psychometric battery to be used to predict women’s ability to estimate their body size, thereby determining whether a more complete, multidimensional account of the attitudinal components that underpin body size estimation is needed.

### 1.5 Current study

The present study was designed to extend and examine the attitudinal-perceptual framework of body size estimation by addressing two interrelated aims.

First, we sought to examine the internal structure of attitudinal body image in greater detail using network analysis on data from a sample of non-clinical adult women. Network analysis has become increasingly influential in psychology (Robinaugh et al., 2020) as it focuses on the connections between elements, such as symptoms, behaviours, or traits, rather than treating them as wholly independent or assume they all reflect a single underlying cause (e.g., Borsboom, 2017; Cramer et al., 2010). Applied to body image, this method allows us to move beyond broad summary factors and instead map how eating pathology, body dissatisfaction, mood, sociocultural pressures, and interoceptive sensibility are organized as a psychological system.

Methodologically, network analysis builds on graph theory. Nodes represent individual elements (e.g., symptoms, behaviours, or questionnaire items), and edges represent the statistical associations between them. Analysing the structure of these networks makes it possible to identify, key nodes (influential elements), communities (clusters of related elements), and global network properties such as stability or resilience (Borgatti et al., 2009; Borsboom et al., 2021).

Our second aim was to test how these network-derived components integrate into the established two-component model of body size estimation as illustrated in Figure 1. To achieve this, we used exploratory factor analysis to recover latent dimensions corresponding to the network communities and entered these as predictors, alongside actual BMI and age, into regression models of self-estimated body size.

## 2 Method

### 2.1 Study design

This study employed a cross-sectional design to investigate the ability of perceptual and attitudinal body image to account for women’s self-estimates of body size. Attitudinal body image was indexed by: interoceptive sensibility, body dissatisfaction, eating disorder pathology, depression, and anxiety. Data collection was conducted online using self-report questionnaires and a computerized task based on the Method of Adjustment (MoA) paradigm (Cornelissen et al., 2017, 2022).

### 2.2 Participants

#### 2.2.1 Recruitment and inclusion criteria

Participants were recruited via social media advertisements between February and May 2023. Advertisements were non-targeted except for limiting participation to Norwegian-speaking women aged 16 to 65 years. To ensure diversity in age representation, targeted recruitment strategies via Facebook were implemented in the later stages of the campaign to increase participation among women aged 35–45 years. To mitigate recruitment bias, study advertisements were framed broadly around women’s health rather than body image concerns.

#### 2.2.2 Demographics

A total of 351 adult women consented to take part in the study. However, 82/351 participants provided incomplete datasets and were excluded from further analysis. The final sample consisted of 269 women (*M* = 37.26 years, *SD* = 11.53 years, range: 16–64 years) who provided complete datasets which contained all demographic, psychometric and psychophysical test responses. A sample size of *N* = 250 to 350 is typically considered sufficient for a network with 20 or fewer nodes (Constantin and Cramer, 2020), which is consistent with our data. The sample was predominantly Norwegian (~90%), with a small proportion reporting other European ethnicities (~12%). BMI distribution was slightly skewed towards lower BMI categories, with a mean BMI of 25.96 (SD = 5.31). Detailed demographics are presented in Figure 2 and Table 1.

**Figure 2:**
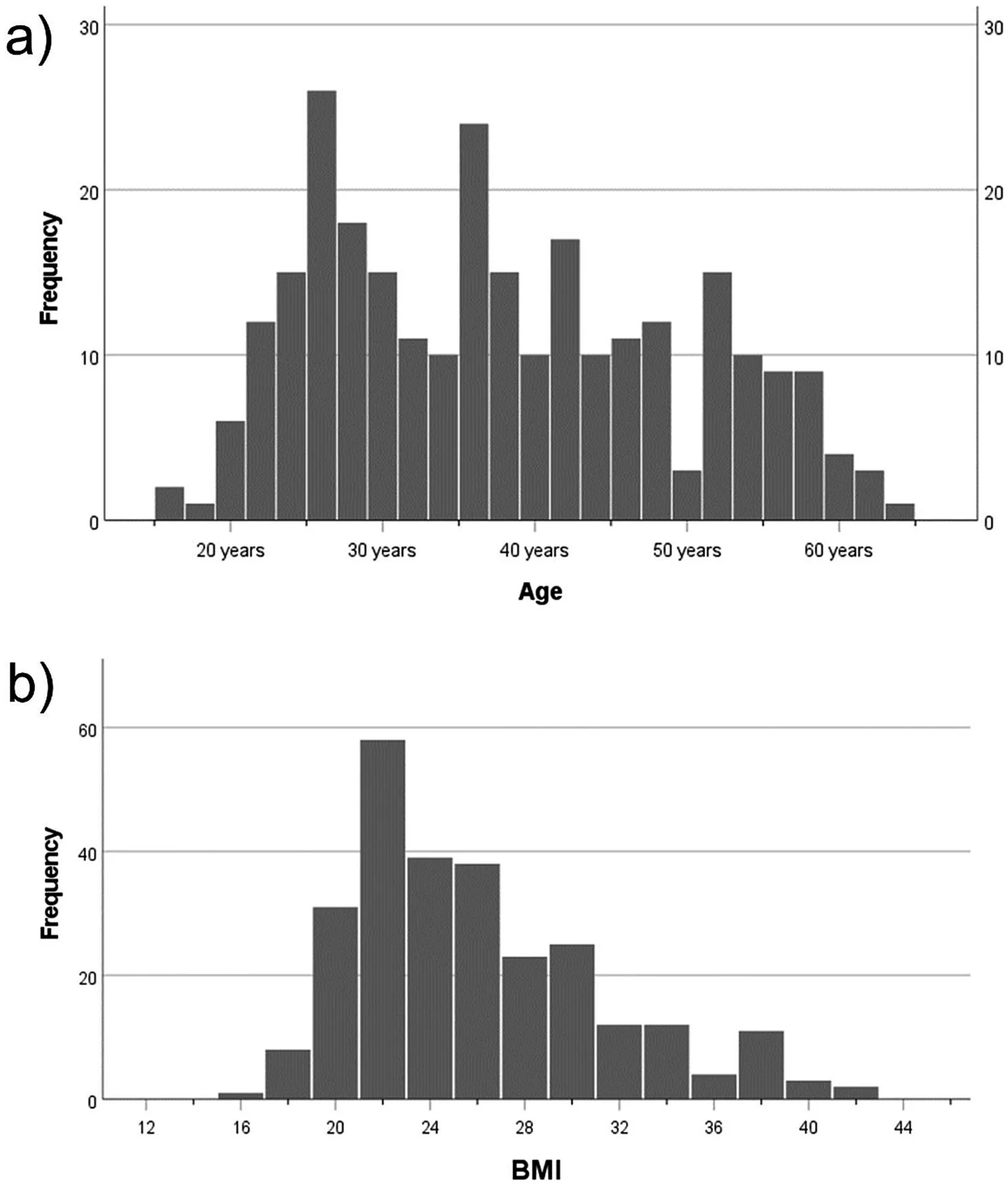
Frequency plots of **(a)** Age and **(b)** BMI in the 269 participants.

**Table 1:** Sample BMI category distribution (*n* = 269).

BMI category	BMI range	*n*	%
‘Underweight’	<18.5	7	2.6%
‘Healthy weight’	18.5–24.9	132	48.7%
‘Overweight’	24.9–30	75	28.1%
‘Obese’	>30	55	20.6%

### 2.3 Variables and measures

#### 2.3.1 Interoceptive sensibility

*The Multidimensional Assessment of Interoceptive Awareness, Version 2* (MAIA-2; Mehling et al., 2018) was used to measure interoceptive sensibility. This 37-item questionnaire is divided into the subscales ‘Noticing,’ ‘Not-Distracting,’ ‘Not-Worrying,’ ‘Attention Regulation,’ ‘Emotional Awareness,’ ‘Self-Regulation,’ ‘Body Listening,’ and ‘Trust.’ Each item is presented as a statement, with agreement measured on a 0 (‘Never’) to 5 (‘Always’) Likert scale. Responses were scored on a 6-point Likert scale, with higher scores indicating greater interoceptive sensibility. A Norwegian translation of the MAIA-2 (Fiskum et al., 2023) was used.

#### 2.3.2 Perceptual body image

*The Method of Adjustment Task (MoA-task)* by Cornelissen et al. (2017, 2022) was used to measure perceptual body image disturbances. The task is computer-based and displays a female CGI-avatar at a random body size (BMI) along with a slider bar for adjusting the avatar’s size (from BMI 12 to 45). In 20 trials, participants are instructed to modify the avatar’s size to reflect their perceived body size, which prompts the next trial. The average chosen body size (in BMI units) can then be calculated from these trials for each participant. The regression slope between these self-estimates of body size and participants’ actual BMI across the sample constitutes the estimate of contraction bias for the sample, and this represents the perceptual body image component for this study.

#### 2.3.3 Attitudinal body image

Attitudinal body image was measured using two measures.

(1) The Norwegian translation of the *Body Shape Questionnaire short form* (BSQ; Evans and Dolan, 1993; Kapstad et al., 2015). This 14-item questionnaire evaluates attitudes and concerns about one’s own body the past 14 days. Items are scored from ‘never’ to ‘always’ (on a Likert scale 0–5), and a higher score indicates greater body dissatisfaction.
(2) Eating disorder pathology was measured using the Norwegian version of the Eating Disorder Examination Questionnaire (EDE-Q 6.0; Fairburn and Beglin, 2008; Rø et al., 2010). The EDE-Q comprises four subscales (Restraint, Eating Concern, Shape Concern, and Weight Concern) with responses scored on a 7-point Likert scale. Clinical cut-off scores of 2.5 (Rø et al., 2015) and 4.0 (Fairburn and Beglin, 2008) were used to identify probable clinical cases.

#### 2.3.4 Internalization of socio-cultural pressures

The 22-item Sociocultural Attitudes Towards Appearance Questionnaire-4 (SATAQ-4; Schaefer et al., 2015) was used to measure internalisation of appearance ideals and appearance related pressures. It assesses attitudes towards one’s appearance using five subscales: two for internalisation of ideals (thin/low body fat and athletic/muscular dimensions) and three for pressures (family, peers, and media dimensions). Each item is rated on a five-point Likert scale, with response options ranging from 1 (definitely disagree) to 5 (definitely agree). Higher scores on each subscale indicate greater internalisation and acceptance of societal appearance ideals, as well as greater pressures from family, peers, and media. The two internalisation sub-scale scores each range from 5 to 25, and the three pressures subscale scores each range from 4 to 16.

#### 2.3.5 Depression

A Norwegian translation of the *Patient Health Questionnaire-9* (PHQ-9; Kroenke et al., 2001; Wisting et al., 2021) was used to measure depression. The PHQ-9 contains nine items assessing depressive symptoms. All items are statements scored on a 0–3 Likert scale, with higher scores indicate increased agreement and more severe depressive symptoms.

#### 2.3.6 Anxiety

Anxiety symptoms were measured using a Norwegian translation of the *General Anxiety Disorder-7* (GAD-7; Johnson et al., 2019; Spitzer et al., 2006). The GAD-7 consists of seven items measuring generalized anxiety symptoms. All items assess the prevalence and frequency of common anxiety symptoms and are scored on a 0–3 Likert scale. Total scores range from 0 to 21, with higher scores indicating more frequent anxiety symptoms.

#### 2.3.7 Body mass index (BMI)

Participants’ actual BMI was calculated using self-reported height and weight. If a participant provided a weight range, the midpoint was used for analysis. A criticism of online testing is the difficulty of verifying participants’ anthropometric details such as height, weight, and BMI. Evidence suggests that individuals tend to over-estimate their height and under-estimate their weight (Connor Gorber et al., 2007; Maukonen et al., 2018), with such estimation errors increasing with participant age and BMI size (Kuczmarski et al., 2001). However, these estimation errors tend to be systematic and can be adjusted by using correction factors (Connor Gorber et al., 2008) which is the procedure we applied in this study.

### 2.4 Procedure

The study was approved by the Norwegian Agency for Shared Services in Education and Research (SIKT; Ref. no. 817144) and the Regional Committee for Medical and Health Research Ethics (REK; Ref. no. 465258). The study was preregistered on the Open Science Framework (OSF; https://osf.io/mxvup/).

Participants were recruited via social media advertisements and physical posters and flyers. The social media advertisement and a QR-code on the posters and flyers directed potential participants to a website containing information about the project.^1^ On this website, a Qualtrics survey link presented the study information and consent form. After consenting, participants answered a few questions to determine their eligibility (age and gender). Eligible participants then created a unique codeword for potential withdrawal requests. The survey included psychometric measures in a randomized order to minimize order effects. Following the survey, participants were directed to the experiment-hosting platform Pavlovia to complete the MoA tasks. Three practice trials were provided before the main trials. To mitigate task fatigue, a brief digit-span task was inserted between the two MoA tasks. After completing all tasks, participants were redirected to Qualtrics for debriefing.

## 3 Data analysis strategy

### 3.1 Data transformation

The mean and sum scores of the psychometric scales were then transformed into *z*-scores in order to standardize the data and facilitate comparisons across different scales. This transformation was necessary because the scales used in the study (e.g., EDE-Q, BSQ-14, GAD-7, PHQ-9, and SATAQ-4) have different scoring ranges and units, making direct comparisons between their scores difficult. After computing the *z*-scores for each scale, we used these standardized values in our subsequent analyses.

### 3.2 Network analysis (NA)

Using R version 4.4.3, we estimated a network that captured various elements of attitudinal body image derived from the psychometric tasks outlined above. We started this process by estimating the number of dimensions (or communities) that underlie the variables in our network using exploratory graph analysis (EGA; Golino and Epskamp, 2017) in conjunction with the walktrap community detection algorithm in the EGAnet package (Golino and Epskamp, 2017). We then computed a graphical Gaussian model (GGM) via the ggmModSelect algorithm implemented in the bootnet R package (Epskamp et al., 2018). In a single stage estimation, the ggmModSelect algorithm automatically searches for the most optimal unregularized GGM. This process involves: (a) generating a range of regularized network structures (100 by default) by varying the Least Absolute Shrinkage and Selection Operator (LASSO) tuning parameter; (b) re-estimating each network without regularisation (non-zero associations after regularisation are freely estimated while associations reduced to zero after regularisation are fixed to zero and therefore remain omitted from the network); (c) selecting an optimal model based on the extended Bayesian information criterion (EBIC); and; (d) further evaluating the selected model by stepwise adding and removing associations until the EBIC is optimized.

The final network structure includes study variables (depicted by nodes) and their pairwise associations (depicted by edges connecting the nodes together). The edges are weighted by partial correlation coefficients (with stronger relationships depicted by wider and more saturated edges). This means that: (a) edges in the network represent pairwise associations after controlling for the influence of all other variables in the network; and (b) edges in the network are non-directional, and no causal inferences can be drawn. To provide a further visual element to our network, we coloured nodes based on cluster membership from the EGA and included a corresponding figure legend.

In addition to the network analyses outlined above, we also identified potentially important relationships between nodes belonging to separate clusters by computing network loadings using the net.loads function in EGAnet (Golino and Epskamp, 2017). We requested revised loadings (default) and identified cross-loadings that are substantively meaningful (> 0.10; Christensen et al., 2021). We also used the centralityPlot function in qgraph (Epskamp et al., 2012) to compute indices of centrality: *Node strength* refers to the sum of the absolute edge-weights between a node and all other nodes to which it is connected in the network (i.e., a measure of how strongly a node is connected to other nodes in the network); *Node closeness* is calculated as the inverse of the average shortest path length from a node to all other nodes in the network (i.e., a measure of how close a node is to all other nodes); and *Node betweenness* quantifies a node’s importance based on how often it lies on the shortest paths between other node pairs in the network (i.e., a measure of a node’s capacity to play a “bridge spanning” role). However, as some of these centrality estimates (i.e., closeness and betweenness) are subject to well-documented conceptual and stability issues in psychological networks (Bringmann et al., 2019), we report node centrality in the Supplementary material for exploratory purposes over primary evaluation.

Finally, in order to assess the internal reliability of the network, we followed the recommendations of Epskamp et al. (2018). First, we estimated the accuracy of centrality indices and edge-weights, by drawing bootstrapped confidence intervals calculated by means of “nonparametric” bootstrapping with 2,500 resamples. Then we used the bootnet package to calculate the correlation stability coefficient. This is a measure of how much the network structure is expected to change when some data is removed (e.g., due to dropping participants). Specifically, it represents the estimated maximum proportion of cases that can be dropped while still maintaining a high correlation (typically 0.7 or higher) between centrality measures (like node strength) calculated from the original network and those calculated from the reduced network with 95% probability. A higher CS coefficient indicates greater stability, suggesting the network structure is less sensitive to data removal. While cutoff scores derived from simulation studies are somewhat arbitrary, researchers often use a CS coefficient of 0.25 as the minimum acceptable value, with 0.50 considered as preferable for interpreting centrality (Epskamp et al., 2018).

### 3.3 Re-evaluation of the two-component model of own body size estimation

We used an exploratory factor analysis (EFA) to re-evaluate the factor structure of the variables in our network model and retrieve factor scores for a subsequent regression model examining own body size estimation.

In Mplus 8.8, we conducted an EFA using robust maximum likelihood (MLR) estimation and default oblique (GEOMIN) rotation. Factor retention was explored using eigenvalues (in combination with parallel analysis), goodness of fit statistics for competing models, and model interpretability. The pattern of factor loadings from each model was assessed based on magnitude (≥0.30 was considered meaningful), degree of cross-loading (number of items loading meaningfully on more than one factor; <0.30), and interpretability (Morin, 2023). Given evidence that traditional factor extraction methods such as parallel analysis function comparably to EGA, we expected to find the same number of factors as dimensions in our network (Golino and Epskamp, 2017).

Once a tenable model was identified, we then extracted the factor scores from the EFA and used them as explanatory variables in a multiple regression analysis to model the body size that participants believed they have. Because Mplus does not save factor scores for TYPE = EFA, we re-estimated the retained solution as an exploratory structural equation model (ESEM), specifying the same number of factors and the same rotation and estimator. This reproduces the EFA factor solution, so the saved factor scores correspond to the EFA factors. We then used PROC REG in SASv9.4 (SAS Institute, North Carolina, US) to run a multiple regression analysis in which we accounted for the variance in self-estimates of body size. The explanatory variables included in a simultaneous model were age, actual BMI (corrected for online errors), and the factor scores from the ESEM. Explanatory variables were retained in the final model if they account for a significant proportion of variance in self-estimated body size (*p* < 0.05).

## 4 Results

### 4.1 Descriptive statistics for psychometric data

Participants completed several self-report measures as part of the study: The EDE-Q to assess eating pathology, the BSQ-14 to assess body image concerns, the GAD-7 to assess anxiety symptoms, and the PHQ-9 to assess depressive symptoms. Each measure was scored using their respective standard procedures, with higher scores indicating greater levels of pathology or symptoms. The ranges, mean scores, standard deviations, clinical cut-off points, percentage of participants with scores ≥ clinical cut-off points, and Cronbach’s alphas are presented in Table 2. All measures showed good to excellent internal reliability (*α* = 0.85–0.96) in line with past literature.

**Table 2:** Range, mean scores, clinical cut-off points, and Cronbach’s alpha of all measures.

Measure (variable)	Measure range (in sample)	Mean score (SD)	Clinical cut-off point	Sample w/scores ≥ clinical cut-off	Measure *α* (sample *α*)
Interoceptive sensibility (MAIA-2)	1–6 (1.57–4.92)	3.41 (0.64)	—	—	0.85 (0.87)
Eating disorder pathology (EDE-Q)	1–7 (1.30–6.70)	3.08 (1.31)	4* / 2.5**	26.39%* (57.25%**)	0.94 (0.95)
Body dissatisfaction (BSQ-14)	1–6 (1.00–6.00)	3.16 (1.41)	56.8	32.34%	0.91–0.96 (0.92)
Anxiety (GAD-7)	1–4 (1.00–4.00)	2.18 (0.76)	10	36.10%	0.88–0.93 (0.90)
Depression (PHQ-9)	1–4 (1.00–4.00)	2.07 (0.68)	10	43.49%	0.86–0.92 (0.88)
Sociocultural pressure (SATAQ-4)	1–5 (1.00–4.40)	2.46 (0.74)	—	—	0.70–0.96 (0.90)
BMI	− (15.99–41.95)	25.92 (5.31)	—	—	—

^*^Using clinical cut-off score by Fairburn and Beglin (2008). **Using clinical cut-off score by Rø et al. (2015) based on Norwegian populations.

A disproportionately large number of participants had scores on one or more measures passing the clinical cut off point. The highest percentage was depressive symptomology where the clinical cut-off was set to a sum of 10 points. For reference, clinical guidelines indicate that a score ≥ 5 can indicate ‘mild’ depression, ≥ 10 ‘moderate’ depression, and ≥ 15 ‘moderately severe’ depression, and ≥ 20 ‘severe’ (Kroenke et al., 2001). For participants passing a clinical threshold for the EDE-Q (eating disorder pathology), the numbers varied substantially based on what clinical threshold was applied. If using a global EDE-Q score of 4 as a clinical cut-off point, 26.39% of participants had clinical rates of eating disorder pathology. If using 2.5 as a cut-off point, as recommended in a Norwegian context (Rø et al., 2015) this number increased to 57.25%.

Finally, Supplementary Figure S1 shows the inter-correlations between MAIA interoceptive sensibility measures, and EDEQ, BSQ, GAD, and PHQ. Consistent with prior literature (e.g., Badoud and Tsakiris, 2017; Todd et al., 2019), this shows a consistent pattern of negative correlations between interoceptive sensibility and attitudinal body image: broadly, as interoceptive sensibility increases, so negative attitudinal body image decreases.

### 4.2 Network analysis

Figure 3A shows the optimized network structure, with the nodes colour coded according to the four clusters identified by the EGA. Cluster 1 (red) comprises all 4 EDEQ subscores, BSQ, SATAQ athletic internalization, SATAQ thinness internalization, and SATAQ media pressure. We characterise cluster 1 as primarily representing body image (BI). Cluster 2 (light green) comprises MAIA noticing, MAIA attention regulation, MAIA emotional awareness, MAIA self-regulation, and MAIA body listening. Cluster 2 therefore primarily characterizes interoceptive sensibility (IS). Cluster 3 (cyan) comprises MAIA not distracting, MAIA not worrying, MAIA trust, GAD and PHQ. Cluster 3 appears to be primarily related to mood. Finally, cluster 4 (lilac) comprises SATAQ family and peer pressures and therefore represents internalization of family and peer pressure to be thin (SATAQfp). Solid black edges indicate positive partial correlations while dashed black edges indicate negative ones.

**Figure 3:**
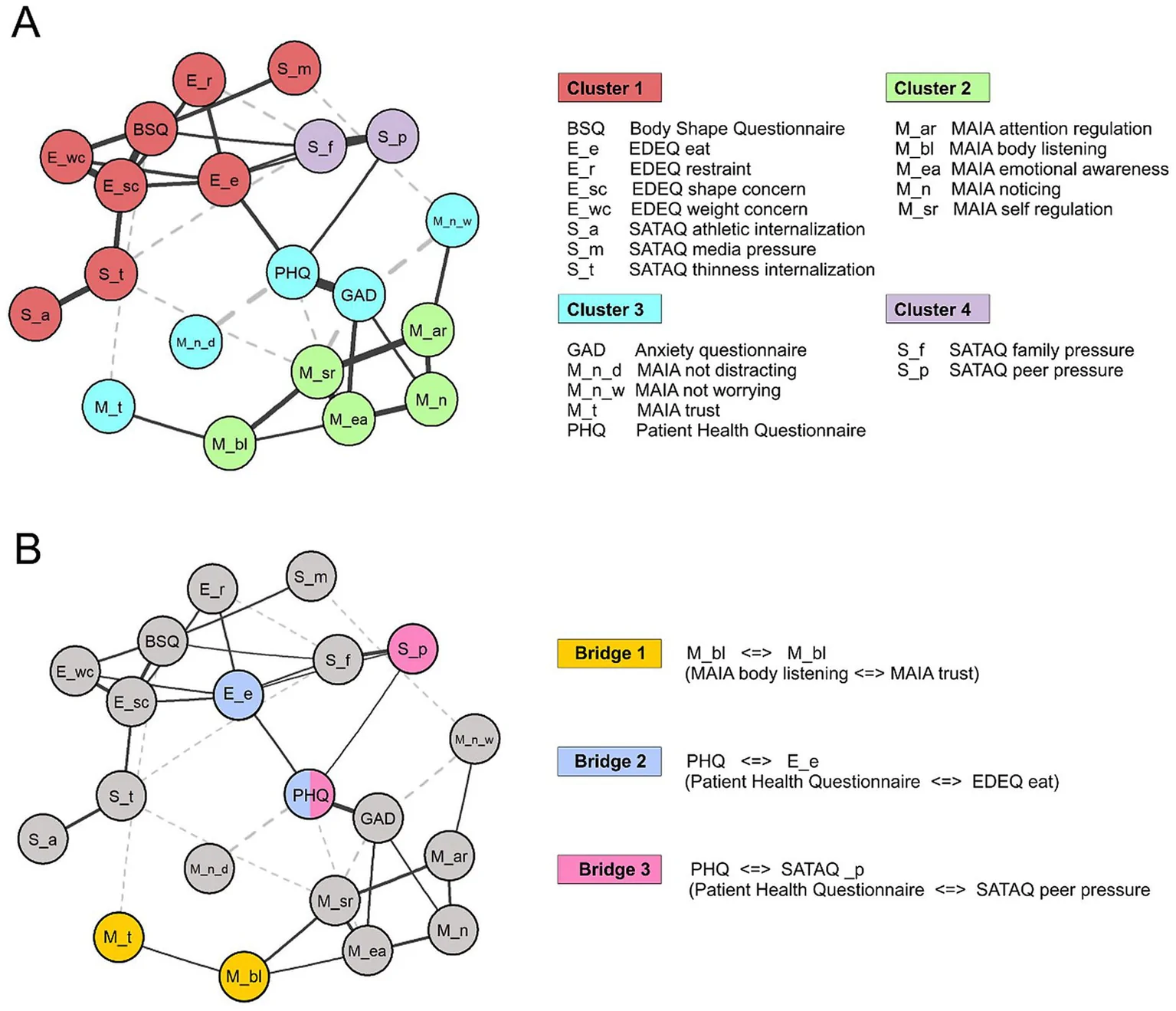
Output from network analysis. **(A)** Optimised network structure, with nodes colour-coded by the four clusters identified by exploratory graph analysis (see legend). Solid edges denote positive and dashed edges negative partial correlations; edge width and saturation are proportional to the size of the partial correlation. **(B)** The same network with the three bridge nodes highlighted: Bridge 1 (yellow), MAIA body listening and MAIA trust; Bridge 2 (blue), PHQ and EDEQ eat; Bridge 3 (pink), PHQ and SATAQ peer pressure (see text for details).

Figure 3B shows the bridge nodes between clusters, coloured in yellow, mauve, and pink. Based on network cross-loadings (CL) having a value > 0.1, we found that EDEQ eat (CL = 0.139 & 0.175), MAIA body listening (CL = 0.104), and PHQ (CL = 0.109) were three candidate bridge nodes. To work out which specific relationships might be of interest (i.e., actual bridges), we examined the partial correlation matrix. This showed that EDEQ eat bridged with PHQ (Cluster 3, *r* = 0.215) and, separately, SATAQ peer pressure (Cluster 4, *r* = 0.131). In addition, MAIA body listening bridged with MAIA trust (*r* = 0.161). These three bridges (MAIA body listening ↔ MAIA trust [*r* = 0.161]; EDEQ eat ↔ PHQ [*r* = 0.215]; PHQ ↔ SATAQ peer pressure [*r =* 0.131]) are represented by the dumbbell pairings in Figure 3B. In short, the IS community primarily connects to the interoceptive component of the Mood community through bridge 1, the mood community primarily connects to the BI community through bridge 2, and the mood community also connects to the SATAQfp community through bridge 3.

Centrality indices for node strength, closeness, and betweenness are illustrated in Supplementary Figure S2, and bootstrapped confidence intervals of estimated edge-weights for the estimated network of 20 psychometric scores are shown in Supplementary Figure S3.

Finally, we assessed the internal reliability of the network in Figure 3A, following Epskamp et al. (2018), by calculating the correlation stability coefficients for the centrality measures and edge weights. The maximum drop proportions to retain correlations of 0.7 in at least 95% of the samples were: strength = 0.595, closeness = 0.362, betweenness = 0.516, and edge weight = 0.674, all of which suggests reasonable internal reliability. This is illustrated in Supplementary Figure S4.

### 4.3 Re-evaluation of the two-component model of own body size estimation

#### 4.3.1 Exploratory factor analysis (EFA)

In the first EFA model tested (using all 20 variables), five eigenvalues exceeded one. However, the parallel analysis supported the retention of either three or four factors—depending on whether the average eigenvalues (four factors supported; F1 6.96 vs. 1.51, F2 2.51 vs. 1.42, F3 1.54 vs. 1.35, and F4 1.29 vs. 1.28) or 95th percentile eigenvalues (three factors supported; F1 6.96 vs. 1.607, F2 2.51 vs. 1.480, and F3 1.54 vs. 1.401) were used to guide evaluation. In evaluating both models, the four-factor model provided superior model fit (*χ*^2^/*df* = 1.92, CFI = 0.96, TLI = 0.94, RMSEA = 0.06 [0.05, 0.07], SRMR = 0.03) over the three-factor model (*χ*^2^/*df* = 2.54, CFI = 0.93, TLI = 0.90, RMSEA = 0.07 [0.07, 0.08], SRMR = 0.04).

While both the three-factor and four-factor models are defensible, the four-factor model was preferred given the stronger evidence for model fit and correspondence with our EGA results. The only departure from the EGA was that MAIA trust failed to load meaningfully meaningfully on any factor. We therefore removed this variable and re-ran the EFA, which resulted in a final model that provided excellent model fit (*χ*^2^/*df* = 2.21, CFI = 0.96, TLI = 0.93, RMSEA = 0.07 [0.05, 0.08], SRMR = 0.03). In this model, four-distinct factors were evident, reflecting (F1) body image (BI), (F2) interoception sensibility (IS), (F3) SATAQ family and peer pressure to be thin (SATAQfp), and (F4) Mood. There was only one case (out of 57 potential cases) of meaningful cross-loading. However, this cross-loading was not considered problematic given that the target loading (MAIA noticing on F2 [Interoception] *λ* = 0.60) is greater than the corresponding cross-loading (MAIA noticing on F4 [Mood] *λ* = 0.43) by a magnitude ≥ 0.10 (Morin, 2023). The factor loadings are shown in Table 3.

**Table 3:** Geomin rotated loadings.

Variable	F1 (Body image, BI)	F2 (Interoceptive sensibility, IS)	F3 (Family and peer pressure, SATAQfp)	F4 (Mood)
EDEQ shape concern	0.973*	−0.015	−0.011	−0.002
BSQ	0.936*	0.041	0.057	−0.044
EDEQ weight concern	0.909*	0.026	0.075	−0.007
EDEQ restraint	0.813*	0.005	−0.102	−0.067
SATAQ thinness internalization	0.739*	−0.117*	−0.227*	0.049
EDEQ eating	0.710*	−0.053	0.144	0.107
SATAQ media pressure	0.454*	0.051	0.119	0.076
SATAQ athletic internalization	0.324*	0.035	−0.175	0.135
MAIA emotional awareness	−0.012	0.844*	−0.034	0.403*
MAIA self-regulation	−0.044	0.782*	0.063	−0.085
MAIA attention regulation	0.071	0.711*	0.018	−0.045
MAIA noticing	−0.003	0.604*	−0.064	0.430*
MAIA body listening	−0.002	0.570*	0.084	−0.024
SATAQ family pressure	0.013	−0.011	0.850*	0.012
SATAQ peer pressure	0.115	−0.029	0.575*	0.123
GAD	−0.009	0.012	0.038	0.919*
PHQ	0.169*	−0.082*	0.102	0.649*
MAIA not worrying	−0.013	0.076	−0.036	−0.401*
MAIA not distracting	−0.128	−0.001	−0.026	−0.343*

Loadings shown in bold and marked with an asterisk (*) are statistically significant at the 5% level.

#### 4.3.2 Multiple regression

In the final step, we carried out a multiple regression analysis of self-estimates of body size. The explanatory variables included in a simultaneous model were age, actual BMI (corrected for online errors), and the factor scores for BI, IS, Mood, and SATAQfp. The final model explained 77.3% of the variance in self-estimated body size. We found significant effects of age, actual BMI (corrected for online error), and Body Image as shown in Table 4.

**Table 4:** Multiple regression output.

Explanatory variable	Regression coefficient	SE	*t*-value	*p*-value
Age	0.20	0.030	6.49	<0.0001
Actual BMI (corrected)	0.74	0.034	21.88	<0.0001
Body image	0.17	0.033	5.21	<0.0001

In each case, age, actual BMI, and Body Image all led to systematic increases in self-estimated body size. We found no evidence for significant two-way interactions, nor any significant contribution to the model from SATAQfp, IS or Mood. Critically, the two-way interaction between actual BMI and Body Image was not significant (*t* = −1.47, *p* = 0.14, *β* = −0.048, SE = 0.032) indicating that the effects of these two explanatory variables are additive. In addition, a test of the slope for actual BMI showed that this was significantly less than 1 (*F*(1,273) = 57.38, *p* < 0.0001), consistent with contraction bias.

## 5 Discussion

The present study sought to explore an integrated model of how perceptual and attitudinal body image components relate to women’s judgments of their own body size. Building on an influential attitudinal-perceptual model of body image (e.g., Cash and Deagle, 1997; Slade, 1988), our aim was not to replace or revise the two-component model per se, but to unpack the relationships between the different potential features contributing to the attitudinal dimension (Forbush et al., 2016; Smith et al., 2018). By integrating network analysis, exploratory factor analysis, and behavioural measures of body size estimation, we tested which psychological processes act as direct predictors of self-estimated body size, and which are merely related within the broader body image system.

Through our analysis, three central findings emerged. First, we robustly replicated contraction bias which is a perceptual effect previously reported in body size judgements. Second, we replicated the independent association of attitudinal concerns with body size estimation. Third, although attitudinal constructs are multidimensional in structure, only the Body Image factor remained as a statistically significant predictor of body size when competing with the three other factors: SATAQfp, Mood, and Interoceptive Sensibility. Thus, only age, actual BMI and Body Image (BI) are directly associated with body size estimation, where BI represents the combination of: body dissatisfaction, concerns around body weight, shape, and eating, and societal pressure to conform to a thin and athletic ideal.

### 5.1 Perceptual body image and contraction bias

Our first major finding was that the slope of self-estimated body size (BMI units) on actual BMI remained significantly less than one, which is a clear replication of contraction bias in perceptual body size estimation: individuals with lower BMIs tend to overestimate their body size while those with higher BMIs underestimate it. As mentioned in the Introduction, current thinking (Jannasch et al., 2025; Loewenstein et al., 2021; Petzschner et al., 2015) suggests that this perfectly normal perceptual bias provides an adaptive, near-optimal strategy for maximizing performance and accuracy in the face of uncertain environmental/sensory input. Furthermore, this is of particular interest in the body image arena because previous research (see, e.g., Brokjøb et al., 2024; Cornelissen et al., 2015) has revealed that women with a history of AN show regression slopes for the relationship between estimated and actual body size > 1 (see sketch graph in Supplementary Figure S5 for illustration). This suggests that they are most accurate at estimating the size of very low weight bodies, but thereafter they increasingly over-estimate body size as body weight increases. In short, they show no evidence of “normal” contraction bias, suggesting a different processing of this perceptual aspect of body size estimation compared to healthy controls (Brokjøb et al., 2024; Cornelissen et al., 2015). Put together with the current findings, this has two important implications. First, deviations in body size perception should not automatically be interpreted as pathological. Second, any theoretical model of body image disturbance must account for both domain-general perceptual constraints and domain-specific psychological factors.

### 5.2 Attitudinal correlates of body size estimation

Beyond perceptual contraction bias, we replicated the finding that attitudinal body image concerns are independently associated with higher self-estimated body size across the entire BMI range. Women who reported greater attitudinal body image concerns, as captured by the body image (BI) factor in this study, consistently judged themselves to be larger than women with the same BMI but lower concerns. As in prior studies (see, e.g., Cornelissen et al., 2015; Brokjøb et al., 2024), these effects were additive rather than interactive: attitudinal concerns were associated with an upward shift of the regression line rather than with a change in its slope. This is consistent with the idea that perceptual and attitudinal factors are independently associated with the body size and shape an individual believes they have.

A key motivation for this study was to explore the adequacy of the original two component model of body-size estimation (illustrated in Figure 1) by asking whether attitudinal body image requires a richer characterization, comprising two or more dimensions, in order to provide an optimal statistical account of body-size estimation. Our analyses succeeded in the sense that the network analysis and factor analysis revealed attitudinal body image to have a rich multidimensional psychological structure comprising four domains. Yet when the outcome of interest is predicting behavioural judgment of body size, a narrower subset of domains appears sufficient—consistent with the two component model.

### 5.3 Interoceptive sensibility

A key aim of the present study was to investigate the role of interoceptive sensibility in body size estimation. Interoceptive sensibility has been repeatedly implicated in attitudinal body image disturbances and eating disorder pathology (Badoud and Tsakiris, 2017; Jenkinson et al., 2018) and has been found to persist even after recovery from eating disorders (Berner et al., 2018; Klabunde et al., 2013). In the present study, however, interoceptive sensibility did not directly predict body size estimation once actual BMI and the Body Image factor were accounted for. Instead, interoceptive variables clustered with mood-related constructs in both the network and factor analyses. Consistent with this, we found a systematic pattern of negative correlations between interoceptive sensibility and attitudinal body image measures, depressive symptomatology and anxiety (Supplementary Figure S1). This pattern of results is therefore consistent with previous literature showing that interoceptive sensibility is associated with attitudinal body image components (cf. Badoud and Tsakiris, 2017; Jenkinson et al., 2018), but the current data do not suggest a clear relationship between interoceptive sensibility and body size estimation per se.

This finding aligns with past research suggesting that self-reported interoceptive awareness often overlaps with emotional appraisal and affective reactivity rather than capturing interoceptive accuracy exclusively (Calì et al., 2015; Desmedt et al., 2022; Murphy et al., 2019). In the present study, several MAIA-2 subscales showed direct links with mood, and, notably, higher scores (indicating better interoceptive sensibility) on facets such as Noticing and Emotional Awareness were associated with elevated anxiety. Although counter-intuitive, this pattern is consistent with the suggestion that some forms of heightened bodily awareness may reflect hypervigilance rather than adaptive bodily attunement. Mensinger et al. (2025), for example, reported that MAIA Noticing, Body Listening, and Emotional Awareness predicted worse eating pathology, highlighting that these dimensions may index maladaptive attentional focus in individuals with body image concerns.

Although the MAIA-2 is widely used, the present findings highlight broader concerns on its construct specificity. In both the network structure and factor solution, interoceptive dimensions did not emerge as a distinct cluster but were embedded within a broader system. This mirrors ongoing critiques that MAIA-2 may not isolate interoceptive processes but instead taps into overlapping affective domains (Mensinger et al., 2025; Vivas-Rivas et al., 2025) and highlights challenges in operationalizing interoceptive constructs due to inconsistent conceptualization across studies and the complex, multidimensional nature of interoception (Rominger et al., 2025; Rogowska et al., 2023). Therefore, an obvious solution that is empirically relevant to the present question: “what size do I think I am?” and to directly test the role of interoceptive function would be to repeat this study using direct measures of interoceptive accuracy, such as heart-beat and/or gastrointestinal vibration detection (e.g., Garfinkel et al., 2015; Verdonk et al., 2026).

### 5.4 Strengths and limitations

A key strength of the present study lies in its multimethod approach, combining behavioural, psychometric, and multivariate modelling techniques. The inclusion of interoceptive measures further extended prior work, and the relatively large sample allowed robust replication of core perceptual and attitudinal effects. Together, these features enhance both the methodological rigour and ecological validity, strengthening confidence in the conclusions.

Several limitations should also be noted. First, the cross-sectional design precludes causal inference about the relationships between perceptual, attitudinal, and interoceptive processes (Maxwell and Cole, 2007). Second, while our sample was relatively large, it was restricted to adult women reducing generalizability to men, adolescents, and clinical populations in whom body image disturbances may operate differently (Murray et al., 2017). Therefore, further studies are required to address these questions. Third, the assessment of interoceptive processes was restricted to interoceptive sensibility based on the self-report MAIA-2. Although internal reliability was strong, the factorial structure and network placement raised concerns about its construct validity as a distinct measure of interoception (Mehling et al., 2018; Ferentzi et al., 2021). Future studies would benefit from multimethod assessments, incorporating behavioural accuracy tasks and physiological indices, to provide a more comprehensive account (Khalsa et al., 2018). Finally, this is an online study. A criticism of online testing is the difficulty of verifying participants’ biographical information, including demographic characteristics such as age, gender, occupation, and SES, as well as anthropometric characteristics such as height, weight, and BMI. As mentioned above, previous research suggests that individuals tend to overestimate their height and underestimate their weight (Connor Gorber et al., 2007; Kuczmarski et al., 2001; Maukonen et al., 2018). Importantly, however, these errors tend to be systematic, allowing self-reported anthropometric data to be adjusted using correction factors (Connor Gorber et al., 2008), as was done in the present study. More broadly, studies comparing online and in-person data collection have generally found little or no difference in the quality or validity of the resulting data (Birnbaum, 2004; Epstein et al., 2001; Gumančík et al., 2024).

A further set of limitations concerns recruitment, task administration, and analytic strategy. First, participants were recruited via social media. Although this affords rapid access to a large and geographically diverse sample, it introduces selection bias: individuals who respond to such advertisements may differ systematically from the general population in age, digital engagement, and in their levels of body image concern and their interest in appearance-related topics. Self-selection of this kind may over-represent individuals for whom body image is salient, potentially reducing the representativeness of the sample and the generalizability of the observed findings. Second, the perceptual body size estimation task was administered online, without the standardized viewing conditions, screen calibration, and experimenter oversight typically available in laboratory settings. Uncontrolled variation in display size, resolution, and viewing distance may therefore have introduced additional measurement noise into the perceptual estimates. However, such error would tend to reduce rather than inflate the effects reported here. Finally, the same sample was used both to identify the latent factor structure (via exploratory factor analysis) and to estimate the subsequent regression models predicting body size estimation. Deriving the factor solution and the inferential models from a single dataset risks capitalizing on sample-specific variance and may inflate both apparent model fit and the apparent strength of the retained predictors. The factor structure and the identification of the Body Image factor as the sole direct predictor should therefore be regarded as provisional, and confirmatory factor analysis and predictive modelling in independent samples will be needed to establish their robustness.

Although BMI is a widely used anthropometric index, it conflates fat mass and lean mass and is vulnerable to statistical artefacts such as Simpson’s paradox. Specifically, a plot of muscle mass as a function of body fat is positively correlated across any reasonably large sample of men or women. However, if the data sets are subdivided into narrow ranges of BMI, then the direction of the relationship between muscle and fat mass inverts and becomes negative (Maalin et al., 2021). It is for exactly this reason that individuals can have the same BMI but different body composition (i.e., higher muscle mass with lower body fat, or vice versa), and therefore different body shapes (Mullie et al., 2008; Yajnik and Yudkin, 2004). These limitations can obscure meaningful variation in body composition that may shape both perceptual and attitudinal components of body image. Future work should incorporate direct measures of body composition (e.g., fat mass, skeletal muscle mass, waist-to-hip ratio) to expand the two-component model beyond BMI space and test whether similar perceptual and attitudinal patterns hold when more physiologically precise indices are used.

While the network analysis revealed coherent community structures and robust edge weights and strength, the stability of certain centrality indices, particularly closeness, was below recommended thresholds (Epskamp et al., 2018). This suggests that the overall structure and identification of bridge nodes in particular should be interpreted with some caution. Replication in larger and independent datasets will be essential to confirm the stability of these features.

## 6 Conclusion

Overall, the present findings carry a dual message for theory and practice. Theoretically, network methods illuminate how diverse psychological processes are interwoven, clarifying the patterns of association that may underlie the maintenance of body image disturbances across domains. Practically, the two-component model remains a powerful and accessible framework capable of explaining core variance in body size judgments in the present non-clinical sample. Because the current data are cross-sectional and were drawn from a non-clinical sample, these findings cannot speak directly to clinical populations or to causal maintenance processes, and any clinical or public-health application remains to be established. Nonetheless, by demonstrating the utility of nuanced, multidimensional analyses, the present study helps bridge the gap between scientific insight and models that are intuitive and replicable and may form a foundation that future longitudinal and clinical studies could build on to assess translational potential.

## Data Availability

The raw data supporting the conclusions of this article are not publicly available because the conditions of the ethical approval and of the participants’ consent do not permit sharing of individual-level data. A synthetic dataset that preserves the statistical properties of the original data (means, variances and covariance structure), together with the analysis code, is available on the Open Science Framework at https://doi.org/10.17605/OSF.IO/Y2TN3.

## Footnotes

1
https://site.uit.no/kvinnehelse/
