# Supplementary material for: What size and shape am I?
Source: Front Psychol. 2026 Sep 28;17:1946881. doi: 10.3389/fpsyg.2026.1946881 (PMC13654794; doi:10.3389/fpsyg.2026.1946881)
Supplement: Supplementary file 1 [file Supplementary_File_1.docx]

**SUPPLEMENTARY MATERIALS**

Figure S1 shows a graphical representation of the Pearson correlation matrix between the sub-scale scores for MAIA, the sub-scale scores for EDEQ, as well as BSQ, GAD, and PHQ. The correlation coefficients r = 0.15, and r = -0.15 equate to a significance level in this sample of 279 individuals of p = 0.01.

Figure S2 shows the three centrality indices for node strength, closeness, and betweenness. For the attitudinal body image (cluster 1), EDEQ shape concern and BSQ have the highest strength and closeness, while SATAQ family pressure and EDEQ shape concern have the highest betweenness. For interoception (cluster 2), MAIA self-regulation and emotional awareness have the hight node strength and closeness, while MAIA self-regulation has the highest betweenness. For mood, (cluster 3), PHQ and GAD have the highest strength, closeness, and betweenness, while for SATAQ family and peer pressures (cluster 4), there is a similar pattern for SATAQ family.

Figure S3 shows bootstrapped confidence intervals of estimated edge-weights for the estimated network of 20 psychometric scores. The red line indicates the sample values and the gray area the bootstrapped CIs. Black dots are the bootstrapped means. Each horizontal line represents one edge of the network, ordered from the edge with the highest edge-weight to the edge with the lowest edge-weight. In the case of ties (for instance, multiple edge-weights were estimated to be exactly 0), the mean of the bootstrap samples was used in ordering the edges. The y-axis labels have been removed to avoid cluttering.

Figure S4. Average correlations between centrality indices of networks sampled with persons dropped and the original sample. Lines indicate the means and areas indicate the range from the 2.5th quantile to the 97.5th quantile

Figure S5. Sketch plot to illustrate findings by Cornelissen et al. (2015). Self-estimated body size is plotted as a function of actual body size. Red and blue lines show the regression relationship separately for women with a history of anorexia and healthy controls respectively. Solid and dashed lines separate each group according to PSYCH. See text for details.

Figure S5. Sketch plot to illustrate findings by Cornelissen et al. (2015). Self-estimated body size is plotted as a function of actual body size. Red and blue lines show the regression relationship separately for women with a history of anorexia and healthy controls respectively. Solid and dashed lines separate each group according to PSYCH. The short-dashed black line is the line of equivalence between self-estimated and actual body size (slope = 1), for reference. Healthy controls show regression slopes less than 1: those with BMIs near the population average estimated most accurately, while lower BMIs were over-estimated and higher BMIs under-estimated. This reflects contraction bias. Women with a history of anorexia showed regression slopes greater than 1. Their self-estimates were most accurate at the lowest actual BMIs, but over-estimation increased systematically as BMI increased. This indicates that perceptual body image disturbance associated with eating disorders may persist post-recovery.

The independent influence of PSYCH is shown by parallel regression lines at +1 and -1 PSYCH for both groups. For any given actual BMI, higher PSYCH scores produced larger self-estimates of body size. This pattern was equivalent across healthy controls and women with a history of anorexia — a given increase in PSYCH produced the same increment in self-estimated BMI regardless of group. This is emphasized by the vertical green line from a single value for actual BMI. This line’s upward projection to intersect with the 4 regression lines produces 4 separate self-estimates of BMI. Thus, 4 individuals (2 women with a history of anorexia nervosa and 2 healthy controls) who all have the same actual BMI each make separate self-estimates of their body size (expressed in BMI units).
